# Supplementary material for: A conserved switch controls virulence, sporulation, and motility in C. difficile
Source: PLoS Pathog. 2024 May 13;20(5):e1012224. doi: 10.1371/journal.ppat.1012224 (PMC11115286; doi:10.1371/journal.ppat.1012224)
Supplement: S1 Table — (DOCX) [file ppat.1012224.s001.docx]

**S1_Table.** Bacterial Strains and plasmids

| **Plasmid or Strain** | | **Relevant genotype or features** | **Source, construction or reference** |
| --- | --- | --- | --- |
| **Strains** | |  |  |
| E. coli | |  |  |
|  | DH5α |  |  |
|  | HB101 | F^-^ *mcrB mrr hsdS20*(r_B_^-^ m_B_*^-^) recA13 leuB6 ara-14* *proA2 lacY1 galK2 xyl-5 mtl-1 rpsL20* (conjugation) | B. Dupuy |
| C. difficile | |  |  |
|  | 630Δ*erm* | Erm^S^ derivative of strain 630 | (1) |
|  | RT1075 | 630Δ*erm sigD*::*erm* | (2) |
|  | MC324 | 630Δ*erm* pMC123 | (3) |
|  | MC855 | 630Δ*erm spo0A*::*erm* pMC123 | (4) |
|  | MC1003 | 630Δ*erm spo0A*::*erm* pMC674 | This study |
|  | MC1615 | 630Δ*erm spo0E*::*erm* | This study |
|  | MC1698 | 630Δ*erm spo0E*::*erm* pMC980 | This study |
|  | MC1699 | 630Δ*erm spo0E*::*erm* pMC123 | This study |
|  | MC1968 | 630Δ*erm spo0E*::*erm* pMC1093 | This study |
|  | MC2523 | 630Δ*erm spo0E*::*erm B.subtilis spo0E::spc* | This study |
|  | MC2695 | 630Δ*erm* pMC1093 | This study |
|  | MC2696 | 630Δ*erm* pMC1432 | This study |
|  | MC2697 | 630Δ*erm* pMC1425 | This study |
|  |  |  |  |
| ***B. subtilis*** |  |  |  |
|  | 1A1 | strain 168 lineage | (5) |
|  | 1S143 | MF2339; PY79 *spo0A::kan* | M. Fujita/BGSC |
|  | BKE13640 | Δ*spo0E::erm* | (5) |
|  | MC2261 | 1S143🡪1A1; *spo0A::kan* | This study |
|  | MC2400 | BKE13640🡪1A1; Δ*spo0E::erm* | This study |
|  | MC2401 | pMC1259 🡪MC2400 | This study |
|  |  |  |  |
| **Plasmids** |  |  |  |
|  | pBB1364 | *kan* | (6) |
|  | pCE240 | *C. difficile* TargeTron construct based on pJIR750ai (group II intron, *ermB::*RAM, *ltrA*); *catP* | C. Ellermeier |
|  | pMC123 | *E. coli-C. difficile* shuttle vector; *bla, catP* | (7) |
|  | pMC228 | pMC123 with *spo0E-*targeted intron | This study |
|  | pMC674 | pMC123 s*po0A::*3xFLAG | (4) |
|  | pMC980 | pMC123 *CD3272*-*spo0E* | This study |
|  | pMC1093 | pMC123 *spo0E::*3xFLAG | This study |
|  | pMC1206 | pMSR with *spo0A* homology regions flanking *aad9 (spc)* |  |
|  | pMC1259 | pBB1364 *CD3272-spo0E* | This study |
|  | pMC1352 | pMSR *B. subtilis spo0E::spc* | This study |
|  | pMC1425 | pMC123 *spo0E::*3xFLAG, *rstA::*HA | This study |
|  | pMC1432 | pMC123 *rstA::*HA | This study |
|  | pMSR | Pseudo-suicide plasmid used for allele exchange in *C. difficile* 630; P*tet::CD2571.1 catP* | (8) |
|  | pRT1099 | pMC123 with *aad9* substituted for *catP* | (9) |

**References**

1. H. A. Hussain, A. P. Roberts, P. Mullany, Generation of an erythromycin-sensitive derivative of Clostridium difficile strain 630 (630Δerm) and demonstration that the conjugative transposon Tn916ΔE enters the genome of this strain at multiple sites. *Journal of medical microbiology* **54**, 137–141 (2005).

2. E. Bordeleau, *et al.*, Cyclic di-GMP riboswitch-regulated type IV pili contribute to aggregation of Clostridium difficile. *Journal of bacteriology* **197**, 819–32 (2015).

3. A. N. Edwards, K. L. Nawrocki, S. M. McBride, Conserved oligopeptide permeases modulate sporulation initiation in *Clostridium difficile*. *Infection and immunity* **82**, 4276–91 (2014).

4. M. A. DiCandia, *et al.*, “Identification of functional Spo0A residues critical for sporulation in *Clostridioides difficile*” (Microbiology, 2022).

5. B.-M. Koo, *et al.*, Construction and Analysis of Two Genome-Scale Deletion Libraries for Bacillus subtilis. *Cell Systems* **4**, 291-305.e7 (2017).

6. S. Lee, *et al.*, Efficacy, heat stability and safety of intranasally administered Bacillus subtilis spore or vegetative cell vaccines expressing tetanus toxin fragment C. *Vaccine* **28**, 6658–65 (2010).

7. S. M. McBride, A. L. Sonenshein, Identification of a genetic locus responsible for antimicrobial peptide resistance in Clostridium difficile. *Infection and immunity* **79**, 167–76 (2011).

8. J. Peltier, *et al.*, Type I toxin-antitoxin systems contribute to the maintenance of mobile genetic elements in Clostridioides difficile. *Commun Biol* **3**, 718 (2020).

9. E. B. Purcell, *et al.*, A Nutrient-Regulated Cyclic Diguanylate Phosphodiesterase Controls Clostridium difficile Biofilm and Toxin Production during Stationary Phase. *Infect Immun* **85**, e00347-17 (2017).
