# Supplementary material for: A conserved switch controls virulence, sporulation, and motility in C. difficile
Source: PLoS Pathog. 2024 May 13;20(5):e1012224. doi: 10.1371/journal.ppat.1012224 (PMC11115286; doi:10.1371/journal.ppat.1012224)
Supplement: S2 Table — (DOCX) [file ppat.1012224.s002.docx]

**S2_Table.** Filtered proteins identified in Spo0E-FLAG co-immunoprecipitation

| **Gene Locus** | **-Log P value^a^** | **Log_2_ Intensity/control^b^** |
| --- | --- | --- |
| CD630_12140 (Spo0A) | 8.0 | 1.4 |
| CD630_24670 (LepA) | 7.0 | 1.3 |
| CD630_32710 (Spo0E) | 6.7 | 1.7 |
| CD630_36680 (RstA) | 5.5 | 1.3 |
| CD630_21730 | 5.4 | 1.4 |
| CD630_20070 | 5.3 | 1.4 |
| CD630_29560 | 5.2 | 1.3 |
| CD630_35120 | 5.0 | 1.4 |
| CD630_21230 | 4.5 | 1.3 |
| CD630_03400 | 4.4 | 1.2 |
| CD630_29800 | 4.2 | 1.2 |
| CD630_00511 | 4.2 | 1.9 |
| CD630_08210 | 4.0 | 1.3 |
| CD630_22640 | 3.7 | 1.2 |
| CD630_26460 | 3.2 | 1.3 |
| CD630_34700 | 3.1 | 1.2 |
| CD630_22090 | 2.6 | 1.4 |
| CD630_03410 | 2.6 | 1.2 |
| CD630_05590 | 2.1 | 1.2 |

^a^Negative log of *t*-test between average protein intensities of Spo0E-FLAG and negative control pulldown

^b^Ratio of averaged log_2_ transformed intensities between Spo0E-FLAG and negative control pulldown
