# Supplementary material for: A conserved switch controls virulence, sporulation, and motility in C. difficile
Source: PLoS Pathog. 2024 May 13;20(5):e1012224. doi: 10.1371/journal.ppat.1012224 (PMC11115286; doi:10.1371/journal.ppat.1012224)
Supplement: S3 Table — (DOCX) [file ppat.1012224.s003.docx]

**S3_Table.** Filtered proteins identified in Spo0A-FLAG co-immunoprecipitation

| **Gene locus** | **-Log P value^a^** | **Log_2_ Intensity/control^b^** |
| --- | --- | --- |
| CD630_15790 (PtpC) | 5.5 | 1.5 |
| CD630_12140 (Spo0A) | 5.5 | 1.3 |
| CD630_32710 (Spo0E) | 5.3 | 1.5 |
| CD630_12310 | 5.2 | 1.2 |
| CD630_03410 | 4.9 | 1.2 |
| CD630_35230 | 4.8 | 1.3 |
| CD630_24040 | 4.7 | 1.2 |
| CD630_21230 | 4.6 | 1.3 |
| CD630_P10 | 3.8 | 1.4 |
| CD630_20070 | 3.8 | 1.4 |
| CD630_19320 | 3.8 | 1.2 |
| CD630_13060 | 3.7 | 1.3 |
| CD630_25220 | 3.6 | 1.3 |
| CD630_23980 | 3.6 | 1.3 |
| CD630_05230 | 3.5 | 1.2 |
| CD630_00200 | 3.3 | 1.2 |
| CD630_01500 | 3.2 | 1.2 |
| CD630_35940 | 3.0 | 1.2 |
| CD630_18490 | 2.9 | 1.3 |
| CD630_35460 | 2.9 | 1.2 |
| CD630_19670 | 2.6 | 1.2 |
| CD630_19640 | 2.4 | 1.2 |
| CD630_21800 | 2.4 | 1.2 |
| CD630_12470 | 2.4 | 1.2 |
| CD630_19660 | 1.8 | 1.2 |

^a^Negative log of *t*-test between average protein intensities of Spo0A-FLAG and negative control pulldown

^b^Ratio of averaged log_2_ transformed intensities between Spo0A-FLAG and negative control pulldown
