# Supplementary material for: A conserved switch controls virulence, sporulation, and motility in C. difficile
Source: PLoS Pathog. 2024 May 13;20(5):e1012224. doi: 10.1371/journal.ppat.1012224 (PMC11115286; doi:10.1371/journal.ppat.1012224)
Supplement: S4 Table — (DOCX) [file ppat.1012224.s004.docx]

**S4_Table.** *C.difficile* projected Spo0E interactions with RstA and Spo0A are mutually exclusive

| **Spo0E**  **residue** | **Spo0A**  **residue** | **RstA**  **residue** |
| --- | --- | --- |
| R17 | A87^a^  K108^a^ | — |
| N21 | G89^a^  Q90^a^ | — |
| E25 | K92^a^ | — |
| N46  D42 | N12 | — |
| Y26 | — | E354 |
| E28 | — | K380 |
| I29  E33 | — | K339 |

^a^Amino acid with published mutational phenotype *(8, 18, 19).*

^b^Predicted aligned error (PAE) < 5 Å.
