# Supplementary material for: A conserved switch controls virulence, sporulation, and motility in C. difficile
Source: PLoS Pathog. 2024 May 13;20(5):e1012224. doi: 10.1371/journal.ppat.1012224 (PMC11115286; doi:10.1371/journal.ppat.1012224)
Supplement: S5 Table — (DOCX) [file ppat.1012224.s005.docx]

**S5_Table.** *C. difficile* and *B. subtilis* respective Spo0E-Spo0A projected interactions

| ***C. difficile*** | | | | ***B. subtilis*** | | | |
| --- | --- | --- | --- | --- | --- | --- | --- |
| **Spo0E residue**^b^ | Spo0E region | **Spo0A**  **residue** | Spo0A region | **Spo0E residue**^b^ | Spo0E region | **Spo0A**  **residue** | Spo0A region |
| R17 | α1 | A87^a^  K108^a^ | β4  β5 | R18 | α1 | A87 | β4 |
| N21 | α1 | G89^a^  Q90^a^ | β4-α4  β4-α4 | —^*^ | —^*^ | —^*^ | —^*^ |
| E25 | α1 | Q90^a^ | β4-α4 | R26 | α1 | Q90^a^ | β4-α4 |
| —^*^ | —^*^ | —^*^ | —^*^ | Q40^a^ | α2 | D11 | β1-α1 |
| D42 | α2 | N12 | β1-α1 | D43^a^ | α2 | N12^a^  K108 | β1-α1  β5 |
| N46 | α2 | N12 | β1-α1 | N47 | α2 | N12^a^  E14^a^ | β1-α1  α1 |
| K53 | C-term | E21^a^ | α1 | —^*^ | —^*^ | —^*^ | —^*^ |

^*^Species-specific Spo0E and Spo0A binding sites.

^a^Amino acid with published mutational phenotype *(8, 18, 19).*

^b^Predicted aligned error (PAE) < 5 Å.
