# Supplementary material for: A conserved switch controls virulence, sporulation, and motility in C. difficile
Source: PLoS Pathog. 2024 May 13;20(5):e1012224. doi: 10.1371/journal.ppat.1012224 (PMC11115286; doi:10.1371/journal.ppat.1012224)
Supplement: S6 Table — (DOCX) [file ppat.1012224.s006.docx]

**S6_Table.** Predicted *C. difficile* Spo0A and *B. subtilis* Spo0E interactions

| ***C. difficile***  **Spo0A^a^** | ***B. subtilis* Spo0E** | ***C. difficile***  **Spo0E^b^** |
| --- | --- | --- |
| D11 | Q40 | — |
| N12 | N47 | D42  N46 |
| E21 | — | K53 |
| A87  K108 | R18 | R17 |
| G89  Q90 | — | N21 |
| Q90 | — | E25 |
| K108 | D43 | — |

^a^Predicted aligned error (PAE) < 5 Å.

^b^Spo0E residues expected to bind with *C. difficile* Spo0A
