# Supplementary material for: A conserved switch controls virulence, sporulation, and motility in C. difficile
Source: PLoS Pathog. 2024 May 13;20(5):e1012224. doi: 10.1371/journal.ppat.1012224 (PMC11115286; doi:10.1371/journal.ppat.1012224)
Supplement: S7 Table — (DOCX) [file ppat.1012224.s007.docx]

**S7_Table.** Predicted *B. subtilis* Spo0A and *C. difficile* Spo0E residue interactions

| ***B. subtilis***  **Spo0A^a^** | ***C. difficile***  **Spo0E** | ***B. subtilis***  **Spo0E^b^** |
| --- | --- | --- |
| D11 | — | Q40 |
| N12 | D42 | — |
| N12, E14 | N46 | N47 |
| N12, K108 | — | D43 |
| E21 | K53 | — |
| A87 | — | R18 |
| A87, K108 | R17 | — |
| Q90 | — | R26 |
| S103 | — | A80 |

^a^Predicted aligned error (PAE) < 5 Å.

^b^ Spo0E residues expected to bind with *B. subtilis* Spo0A
