# Supplementary material for: A conserved switch controls virulence, sporulation, and motility in C. difficile
Source: PLoS Pathog. 2024 May 13;20(5):e1012224. doi: 10.1371/journal.ppat.1012224 (PMC11115286; doi:10.1371/journal.ppat.1012224)
Supplement: S8 Table — (DOCX) [file ppat.1012224.s008.docx]

**S8_Table.** Oligonucleotides

| **Primer** | **Sequence (5’🡪3’)^a^** | **Use/locus tag/reference** |
| --- | --- | --- |
| oMC513 | GCGGATCCGACAAAATATAATATTGTTTGATAAAATG | Forward primer for *CD630*_*32710* |
| oMC514 | GACGGATCCCTGTGGGCTATTTGCTTAGG | Reverse primer for *CD630*_*32710* |
| oMC515 | AAAAGCTTTTGCAACCCACGTCGATCGTGAA-AACTCTTCTTGA-GTGCGCCCAGATAGGGTG | *CD630_32710* IBS targeting *spo0E* |
| oMC516 | CAGATTGTACAAATGTGGTGATAACAGATAAGTC-TCTTGAAA-TAACTTACCTTTCTTTGT | *CD630_32710* EBS1 targeting *spo0E* |
| oMC517 | CGCAAGTTTCTAATTTCGGTT-GAGTT-TCGATAGAGGAAAGTGTCT | *CD630_32710* EBS2 targeting *spo0E* |
| oMC2589 | AGGGATCCATCACTAAAATTGTAACAAGTATGATAC | Forward primer for *CD630*_*32720-32710* complement |
| oMC2590 | CAGGAATTCACAGATAATTTACACATCAGAAATAC | Reverse primer for *CD630*_*32720*-*32710* complement |
| oMC2919 | GACCACACCCGTCCTGTGGATCCGCTGATTGAGCTTTAGTTTCTTCTT | Forward primer for *rstA::*HA Gibson into pMC1093 |
| oMC2920 | CCGCCGAAGGAATGGTGCATGCTCAGGCATAATCAGGAACGTCGTATGGGTAACCTCCCATTATTTCTAAGTTTTTGTACATAAATACACC | Reverse primer for *rstA::*HA Gibson into pMC1093 |
| oMC3527 | CTCTACAGCAAAATAGATTGTAGTTCTTCGGATCCAATTATAATTGGGTCTCCAAGTGGG | Forward primer for 3’arm of *spo0E* for assembly into pMSR |
| oMC3532 | CTTGCATGTCTGCAGGCCTCGAGACTGGAGTACATCTTATCTATCTTCCATT | Reverse primer for 3’ arm of *CD630_32710* for assembly into pMSR |
| oMC3745 | TGTTCAGAAGAACCGCCCATAATAATCACCACTATTACAAATTTGTATATTTTTATATAC | Reverse primer for 5’ *CD630*_*32720* |
| oMC3746 | GTATATAAAAATATACAAATTTGTAATAGTGGTGATTATTATGGGCGGTTCTTCTGAACA | Forward primer for 5’ *BSU1364* |
| oMC3747 | GTCTTGCCAGTCACGTTACCTATTTATTTGCATCATATGCTGGCATAAC | Reverse primer for 3’ *BSU1364* |
| oMC3748 | GTTATGCCAGCATATGATGCAAATAAATAGGTAACGTGACTGGCAAGAG | Forward primer for *aad9* (spc cassette) with 5’ *spo0E* homology |
| oMC3749 | CAAAAAAACCCACAACTGTGGGACCCAAAATTGAAAAAAGTGTTTCC | Reverse primer for *aad9* (spc cassette) with 3’ *spo0E* homology |
| oMC3750 | GGAAACACTTTTTTCAATTTTGGGTCCCACAGTTGTGGGTTTTTTTG | Forward primer for *3’ spo0E* |
| oMC4003 | TTCGAGCTCGGTACCCGGGGATCCGCTGATTGAGCTTTAGTTTCTTCTT | Forward primer for *rstA::*HA Gibson into pMC123 |
| oMC4004 | GACCATGATTACGCCAAGCTTGCATGCTCAGGCATAATCAGGAACGTCGTATGGGTAACCTCCCATTATTTCTAAGTTTTTGTACATAAATACACC | Reverse primer for *rstA::*HA Gibson into pMC123 |

**^a^**Restriction sites underlined
