# Supplementary material for: A conserved switch controls virulence, sporulation, and motility in C. difficile
Source: PLoS Pathog. 2024 May 13;20(5):e1012224. doi: 10.1371/journal.ppat.1012224 (PMC11115286; doi:10.1371/journal.ppat.1012224)
Supplement: S9 Table — (DOCX) [file ppat.1012224.s009.docx]

**S9_Table.** Vector and strain construction

| **Vector/Strain** | **Construction details** |
| --- | --- |
| pMC228 | The group II intron of pCE240 was targeted to *spo0E* at nucleotide 75 by splicing PCR using oMC515, oMC516, oMC517, and EBSu as outlined in the TargeTron user manual (Sigma-Aldrich). The group II *CD1558*-targeted intron was subcloned using the BsrGI and HindIII sites into pCE240. A 5.45 kb SphI/SfoI fragment was then cloned as SphI/SnaBI into pMC123. |
| pMC980 | A 963 bp product containing full-length operon *CD630­*_*32720*-*spo0E* to complement the *spo0E* mutant was generated using primers oMC2589 and oMC2590 and cloned into pMC123 using BamHI and EcoRI sites. |
| pMC1093 | A 683 bp fragment encoding *C. difficile spo0E* with a C-terminal 3xFLAG tag driven by the *spo0E* native promoter was synthesized by Genscript and cloned into pMC123 using BamHI and EcoRI sites. |
| pMC1259 | The 963 bp fragment containing *C. difficile spo0E* and upstream region from pMC980 was cloned using BamHI and EcoRI sites into pBB1364 for integration at the *B. subtilis sacA* locus. |
| pMC1352 | Four fragments were cloned by Gibson assembly into pMSR for integration into the chromosome to replace the *C. difficile spo0E* region with *spo0E* of *B. subtilis.* A 5’ *spo0E* homology arm (948 bp) was generated from 630∆*erm* genomic DNA with primers oMC3527/3745, a 257 bp fragment containing *B. subtilis spo0E* and upstream region was generated with primers oMC3746/3747 from *B. subtilis* 1A1 genomic DNA, a spc cassette (*aad9*) was amplified from pRT1099 using primers oMC3748/3749, and a 3’ *spo0E* homology arm was generated from 630∆*erm* genomic DNA with primers oMC3750/3532. Fragments were Gibson assembled into pMSR that was digested with BamHI and XhoI. |
| pMC1425 | A 1815 bp product containing full length *rstA::*HA and its promoter region were amplified from 630∆*erm* using primers oMC2919/2920 and cloned into pMC1093 as BamHI/SphI |
| pMC1432 | A 1815 bp product containing full length *rstA::*HA and its promoter region were amplified from 630∆*erm* using primers oMC4003/4004 and cloned into pMC123 as BamHI/SphI |
