# Supplementary material for: A conserved switch controls virulence, sporulation, and motility in C. difficile
Source: PLoS Pathog. 2024 May 13;20(5):e1012224. doi: 10.1371/journal.ppat.1012224 (PMC11115286; doi:10.1371/journal.ppat.1012224)
Supplement: S1 Fig — (PDF) [file ppat.1012224.s010.pdf]

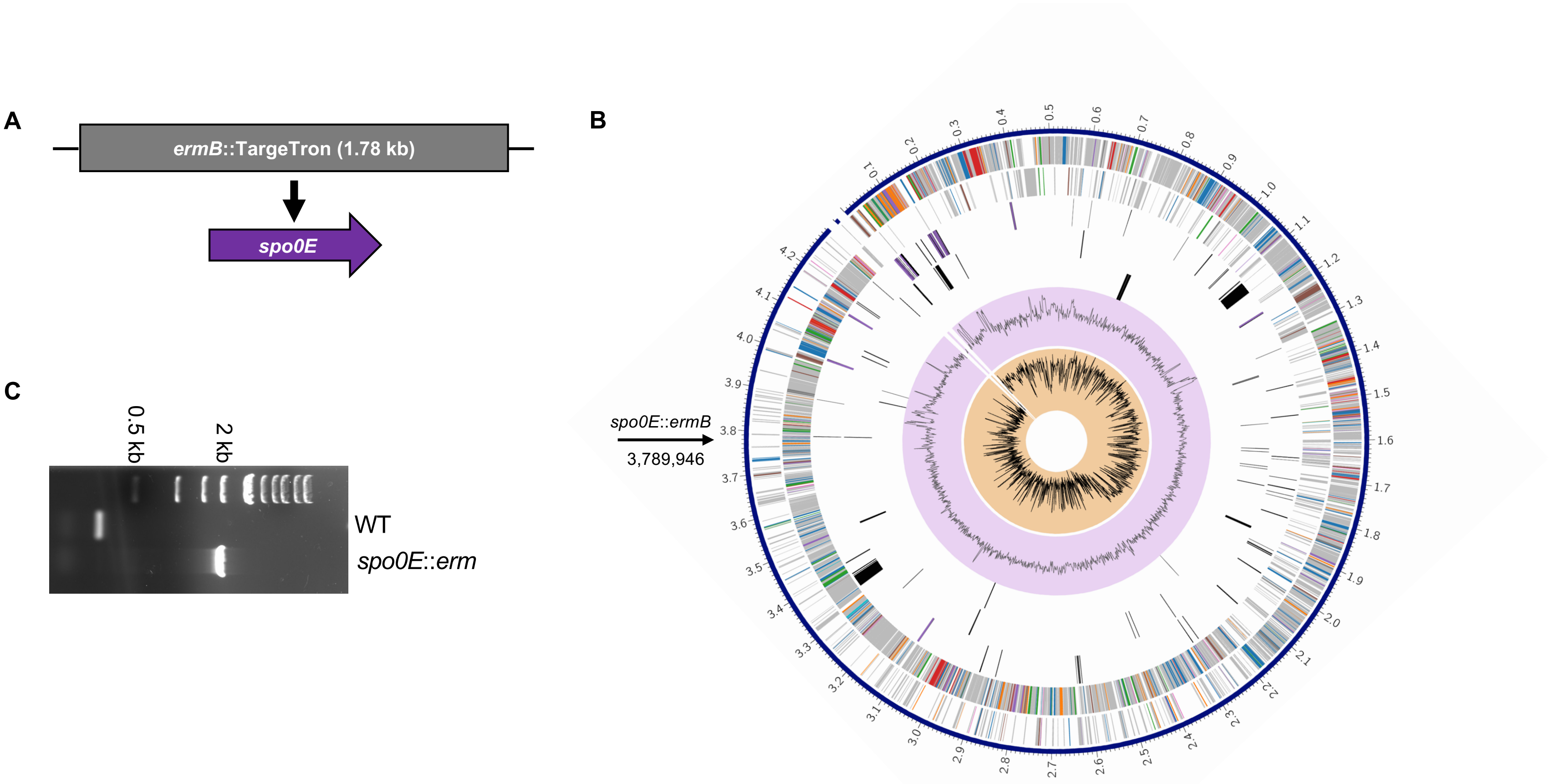

S1_Figure. Construction and confirmation of the *spo0E* mutant. A) The retargeted group II intron (TargeTron) conferring erythromycin resistance (*ermB*) was inserted 59 bp into the coding region of *spo0E*. **B)** Circos plot of *de novo* assembly of the *spo0E* mutant genome using Illumina and Nanopore reads to confirm the TargeTron inserted solely into the *spo0E* locus. Reads were assembled into two contigs (4.29 Mb genome and 7.8 kb endogenous plasmid, respectively) using Unicycler, and the assembly was annotated using Geneious Prime v2022.2.2. Circos plot was generated using PATRIC web resources. Dashes/lines from outermost to innermost ring are: contigs with genomic position, (+) strand CDS, (-) strand CDS, RNA CDS, predicted antimicrobial resistance genes, predicted virulence factors, GC content, and GC skew, respectively. **C)** PCR analysis of 630Δ*erm* (WT) and *spo0E* mutant (MC1615) using primers oMC513/oMC514 to confirm the integration of the ~2 kb intron into *spo0E*. Wild-type product 237 bp; *spo0E::erm* product ~2.2 kb.
