# Supplementary material for: A conserved switch controls virulence, sporulation, and motility in C. difficile
Source: PLoS Pathog. 2024 May 13;20(5):e1012224. doi: 10.1371/journal.ppat.1012224 (PMC11115286; doi:10.1371/journal.ppat.1012224)
Supplement: S2 Fig — (PDF) [file ppat.1012224.s011.pdf]

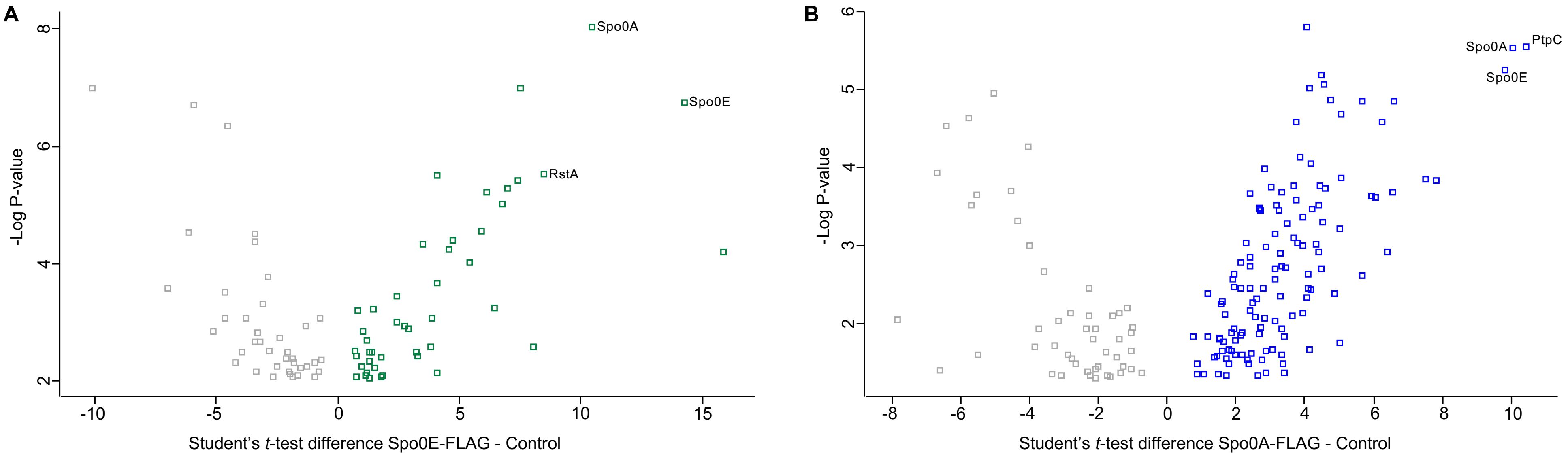


**S2_Figure. Spo0E and Spo0A co-purify with regulators of sporulation, toxin, and motility. A)** Scatter plot of enriched proteins identified in the Spo0E pulldown comparing the mass spec profiles of Spo0E-FLAG (MC1968, green, 45 unique proteins) and vector control (MC324, gray) and **B)** Spo0A pulldown comparing mass spec profiles of Spo0A-FLAG (MC1003, blue, 124 unique proteins) and vector control (MC324, gray). $P \leq 0.05$. Scatter plots generated using Perseus Version 1.6.15.0.
