# Supplementary material for: A conserved switch controls virulence, sporulation, and motility in C. difficile
Source: PLoS Pathog. 2024 May 13;20(5):e1012224. doi: 10.1371/journal.ppat.1012224 (PMC11115286; doi:10.1371/journal.ppat.1012224)
Supplement: S3 Fig — (PDF) [file ppat.1012224.s012.pdf]

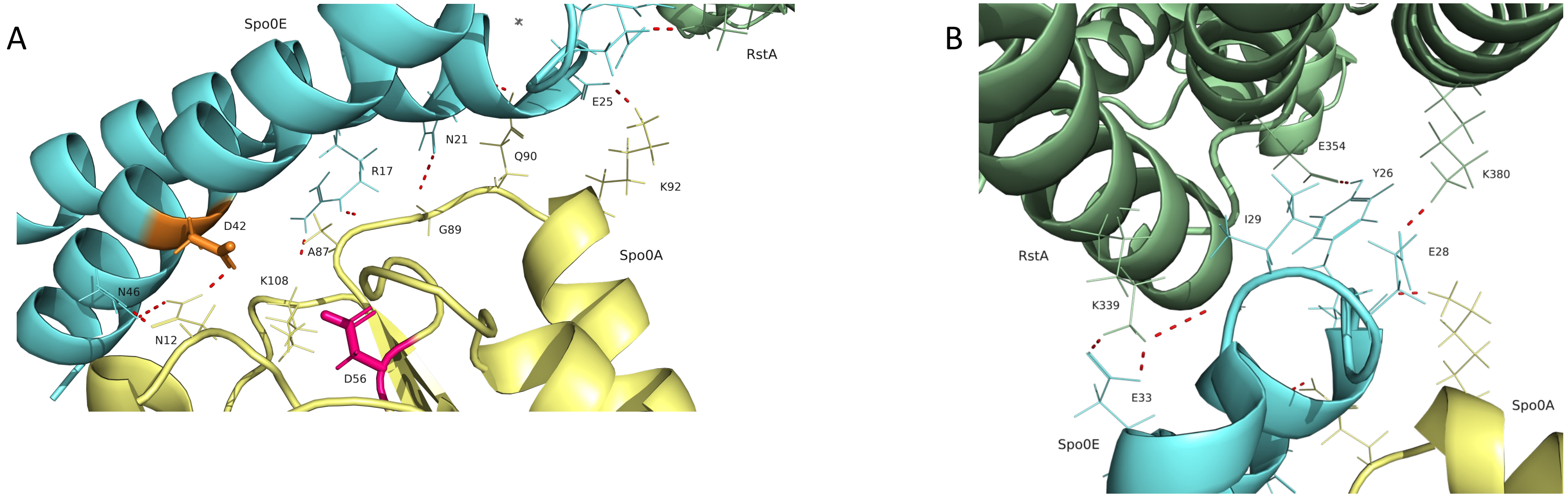

S3_Figure. Predicted *C. difficile* Spo0E interactions with Spo0A and RstA. A) Predicted structural interface of *C. difficile* Spo0A and Spo0E. The Spo0A N-terminal receiver domain (amino acids 5-115) is shown in yellow, with the conserved phosphorylation site (D56) labelled pink. Spo0E is shown in blue with the conserved D42 of the SxxxD motif highlighted in orange. **B**) Structural interface of *C. difficile* RstA and Spo0E. The RstA C-terminal TPR domains are shown in green and Spo0E residues are in blue. Red dashes indicate polar contacts between amino acids. Spo0A Uniprot ID: Q18B74, Spo0E Uniprot ID: A0A7Y0LUW8, edited in PyMol (PyMOL Molecular Graphics System, Version 2.0 Schrödinger, LLC).
