# Supplementary material for: A conserved switch controls virulence, sporulation, and motility in C. difficile
Source: PLoS Pathog. 2024 May 13;20(5):e1012224. doi: 10.1371/journal.ppat.1012224 (PMC11115286; doi:10.1371/journal.ppat.1012224)
Supplement: S4 Fig — (PDF) [file ppat.1012224.s013.pdf]

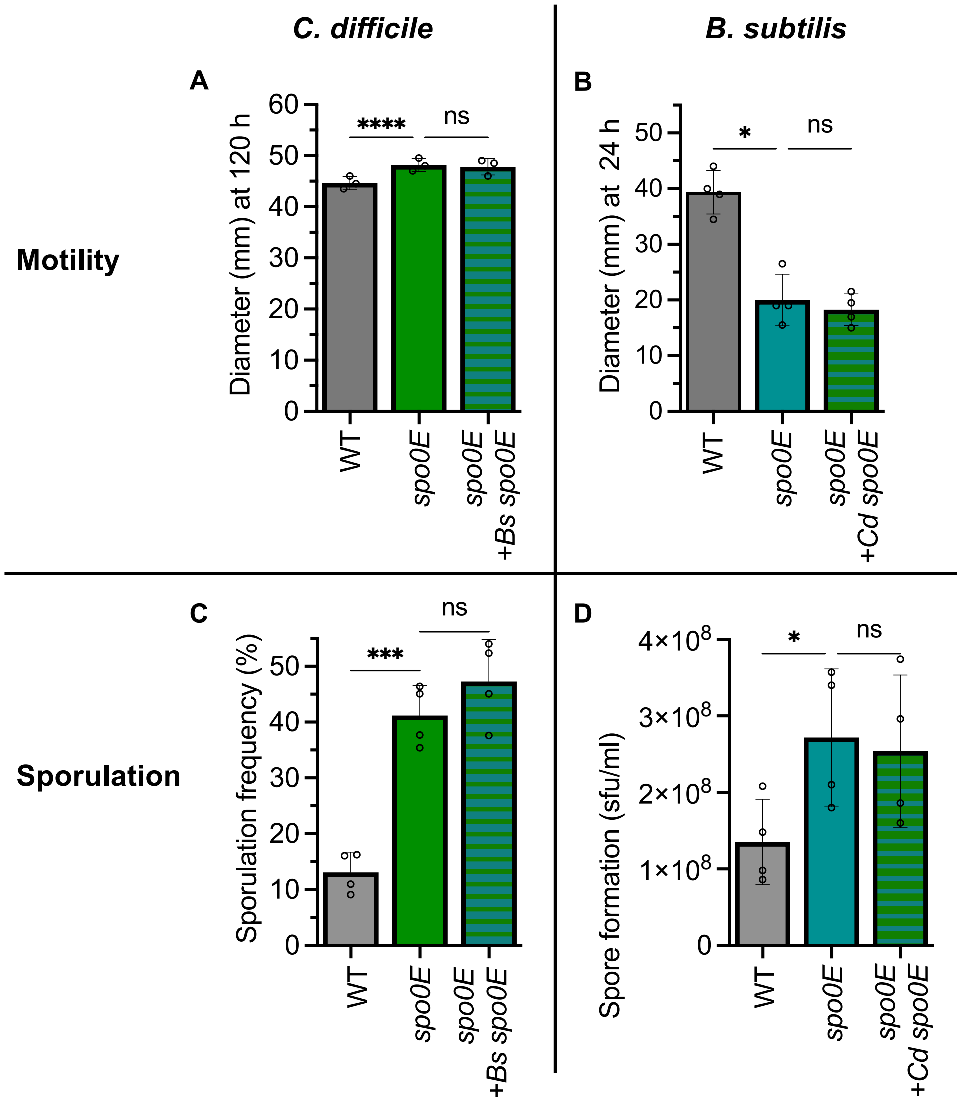

S4_Figure. Spo0E of *C. difficile* and *B. subtilis* do not cross-complement *spo0E* mutant motility or sporulation phenotypes.
Swimming motility of **A)** *C. difficile* WT (630Δ*erm*), *spo0E* mutant (MC1615), and *spo0E* mutant with *B. subtilis spo0E* (MC2523) or **B)** *B. subtilis* IAI (WT), *spo0E* mutant (MC2400), and *spo0E* mutant with *C. difficile spo0E* (MC2401). Active cultures were injected into soft agar and swim diameters measured at 24-120 h, as indicated.
Ethanol-resistant spore formation of **C)** *C. difficile* strains 630Δ*erm* (WT), *spo0E* mutant (MC1615), and MC2523 grown on 70:30 sporulation agar for 24 h or **D)** *B. subtilis* strains IAI (WT), *spo0E* mutant (MC2400), and *spo0E* mutant with *C. difficile spo0E* (MC2401) grown on DSM sporulation agar for 24 h. *spo0A* mutants were used as negative controls to verify elimination of vegetative cells for all assays (not shown). The means and SD of at least three independent experiments are shown. A one-way ANOVA with Dunnett's (A-C) or Holm-Sidak (D) multiple comparisons test were performed; **P* = <0.05, ****P* = <0.001.
