# Supplementary material for: A conserved switch controls virulence, sporulation, and motility in C. difficile
Source: PLoS Pathog. 2024 May 13;20(5):e1012224. doi: 10.1371/journal.ppat.1012224 (PMC11115286; doi:10.1371/journal.ppat.1012224)
Supplement: S5 Fig — (PDF) [file ppat.1012224.s014.pdf]

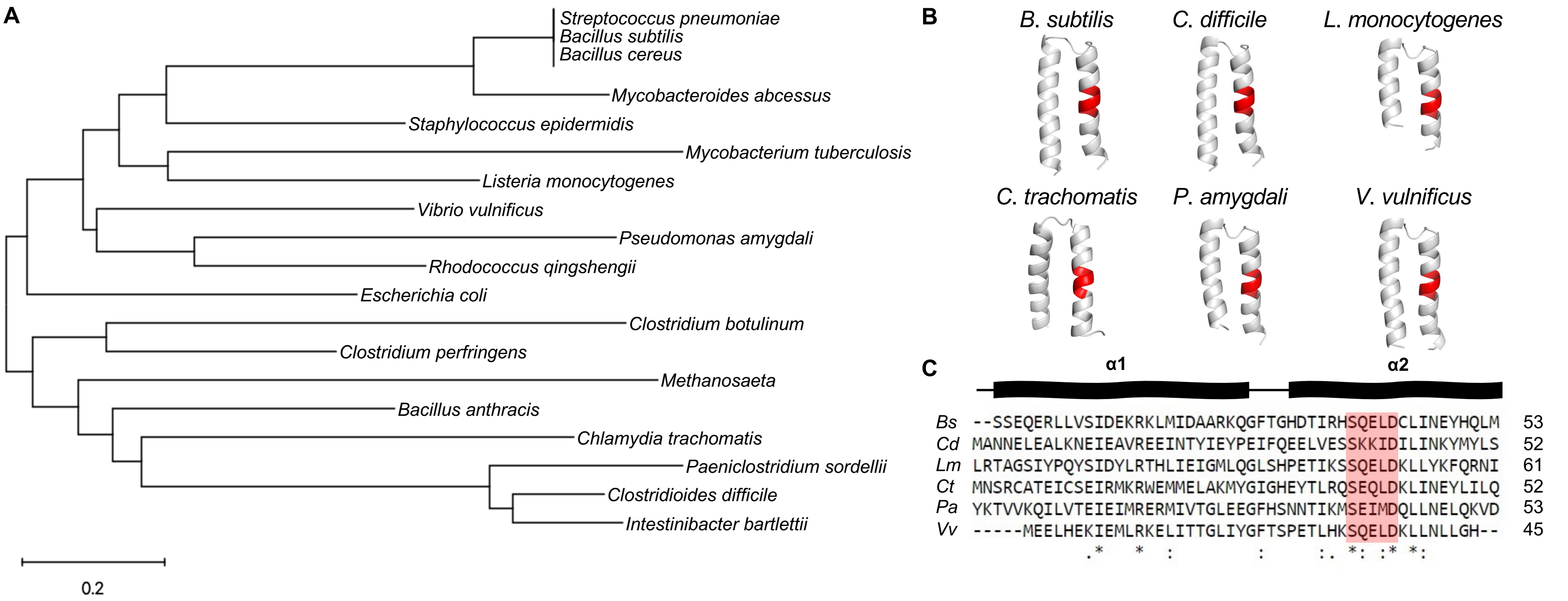

S5_Figure. Spo0E-like proteins are conserved and prevalent in Gram-positive and Gram-negative bacteria. A) Unrooted neighbor-joining tree based on the full amino acid sequence of Spo0E and Spo0E-like proteins. Tree generated using MEGA 11. **B)** The predicted Spo0E 3D structures generated with Phyre2 from representative Gram-positive and Gram-negative species. The residues comprising the signature Spo0E motif (SQELD/SxxxD) are colored red in each structure. Structures were edited in PyMOL (The PyMOL Molecular Graphics System, Version 2.4.0 Schrödinger, LLC). **C)** Multiple sequence alignment of Spo0E and Spo0E-like proteins overlayed against *B. subtilis* Spo0E secondary structure determined by AlphaFold and consisting of two α-helices. Spo0E motif residues are shaded red. Multiple sequence alignment performed using ClustalW.
