# Supplementary material for: A conserved switch controls virulence, sporulation, and motility in C. difficile
Source: PLoS Pathog. 2024 May 13;20(5):e1012224. doi: 10.1371/journal.ppat.1012224 (PMC11115286; doi:10.1371/journal.ppat.1012224)
Supplement: Attachment — Submitted filename: Spo0E Response to Reviewers.docx [file ppat.1012224.s015.docx]

*Editor note:*

The Reviewers were enthusiastic about the finding that Spo0E regulates multiple processes in both C. difficile and B. subtilis. The finding that Spo0E interacts directly or indirectly with Spo0A and RstA in C. difficile is an important contribution, but Reviewers 1 and 3 request additional verification of these mass spectrometry-based co-IP experiments either in the form of validating the AlphaFold-predicted interactions or through another experimental method. They raised additional points regarding alternative interpretations of the data that can be readily addressed through textual changes. If these items are addressed, I hope to make a final decision without needing to send the manuscript out for review again.


Reviewer's Responses to Questions

**Part I - Summary**
Please use this section to discuss strengths/weaknesses of study, novelty/significance, general execution and scholarship.

Reviewer #1: This manuscript describes new phenotypical observations and mechanistic explanations of how the small protein, Spo0E, impacts sporulation, motility, and toxin production in C. difficile. TargeTron disruption of spo0E was generated, validated by sequencing, and complemented to demonstrate that sporulation frequency and TcdA+TcdB toxin is negatively impacted by Spo0E under lab conditions, and it has a modest negative impact on motility. The mutant displays an enhanced virulence phenotype in an animal model of infection. To identify proteins that interact with Spo0E that could explain phenotypes beyond sporulation, Co-IP was conducted. RstA, a transcription factor, was a top candidate. AlphaFold modeling was used to predict interactions between Spo0E, RstA and Spo0A. Residues known to confer interactions between Spo0E and Spo0A from prior studies in Bacillus subtilis were identified in the model’s protein-protein interface, providing confidence in the Spo0E-Spo0A prediction. The role of Spo0E in B. subtilis motility was investigated, as it had not been questioned before, and indeed found to impact this characteristic. However, cross-complementation studies (placing C.diff spo0E into B.sub, and vice versa) found that they could not substitute for one another. Yet, spo0E-like proteins are identified among a variety of bacteria, based on sequence and structural predictions, and therefore indicate these small proteins are likely to be important regulators.
The primary weakness of this study is seen in its reliance on Alphafold predictions between Spo0E and RstA. No evidence is provided to support the notion that Spo0E and RstA actually interact. Nevertheless, the manuscript is easy to follow, the included experiments are technically sound, and the findings are highly significant.

Reviewer #2: The manuscript by DiCandia et al. examines the phosphatase Spo0E in Clostridium difficile. In B. subtilis, Spo0E represses the function of Spo0A, the master regulator of entry into sporulation, but the function of Spo0E in C. difficile is not known. The authors report that in C. difficile, spo0E also represses sporulation but unexpectedly discovered that Spo0E also increases motility and toxin production. Interestingly, an effect of Spo0E on motility (albeit, the opposite effect, but still Spo0A-independent) was conserved and previously overlooked in B. subtilis. Most strikingly, the authors report that Spo0E-like proteins are broadly conserved in bacteria, suggesting that a similar regulatory mechanism exists in other, non-sporulating, cells.

Reviewer #3: Overall this is a well written manuscript describing the important role of Spo0E in regulating sporulation, virulence, toxin production and motility. This is mostly a descriptive study showing the importance of Spo0E in regulating these phenotypes. The authors use Co-IP mass spec to identify interacting partners of Spo0E and Spo0A. There is not significant follow-up demonstrating the importance of these interactions.

**Part II – Major Issues: Key Experiments Required for Acceptance**
Please use this section to detail the key new experiments or modifications of existing experiments that should be absolutely required to validate study conclusions.

Generally, there should be no more than 3 such required experiments or major modifications for a "Major Revision" recommendation. If more than 3 experiments are necessary to validate the study conclusions, then you are encouraged to recommend "Reject".

Reviewer #1: Major Comments:

1. Foremost, the AlphaFold prediction needs to be validated experimentally by some method, preferably showing the predicted interface between Spo0E and RstA is true.	In addition to the Spo0E and Spo0A pulldowns and mass spec analyses, we now confirm by western blot analysis the interactions between Spo0E and RstA using antibodies to each of the proteins in the new Figure 4.

2. Line 169; I disagree with the statement, “the number of spo0E mutant bacteria present in cecum was similar to the WT strain.” It seems there are obviously less bacteria in the distribution of mutant-infected hamsters, compared to the wildtype infected animals. This is interesting given the enhanced virulence of the mutant, which is likely due to elevated toxin production.	We understand the concern and have changed the statement to read “the number of spo0E mutant bacteria present in the cecum was less than the WT strain, but did not achieve statistical significance..”

3. It appears from figure 3 that Spo0E interacts with a monomer of RstA. Is it known whether RstA needs to form a dimer to bind DNA, like most RRNPP transcriptional regulators with a DNA-binding domain?	Some RRNPP can bind DNA as dimers or tetramers, depending on their activation state and cofactor binding. It is likely that RstA binds DNA as a tetramer, but we do not have clear data to support this.

Would the Spo0E-RstA interaction be possible if RstA were in a dimeric complex?	There does not appear to be strong predicted interactions (Alphafold) between Spo0E and dimeric RstA, though it is possible that RstA binding to a co-effector would change its confirmation to facilitate interactions in multimeric forms. In the Spo0E pulldowns, RstA was found in the monomer and dimer forms. Unfortunately, we do not know the identity of the RstA coeffector to further investigate this mechanism.

If RstA were to require dimer formation to bind DNA (likely) might the mechanism of Spo0E work by preventing RstA dimer formation? Is there a way to easily test whether Spo0E impacts RstA dimerization?	As the reviewer suggests, Spo0E may prevent RstA-DNA interactions, but we have no direct evidence for this. The low abundance of RstA and the missing co-effector make this difficult to test, but these are directions we are attempting to pursue.

4. As Spo0E was found to interact with both Spo0A and RstA by Co-IP, and because RstA is reported to impact sporulation by an unknown mechanism, a model is put forth (line 206, Fig. S7) that Spo0E serves as an intermediate between the two regulatory proteins. It could be argued that Spo0E doesn’t need to physically bridge RstA to Spo0A for RstA to have an impact on sporulation. Perhaps Spo0E interacts with one partner at a time and the effect on sporulation is merely an outcome of whether Spo0E is free to engage Spo0A. I suggest including this alternative scenario that Spo0E may not need to function as intermediate between RstA and Spo0A.	We initially considered that Spo0E would switch between RstA and Spo0A as interacting partners. While it may in some cases, we found some RstA pulled down with Spo0A in ¾ of the pulldown assays, indicating that it can bind to both proteins simultaneously. As suggested, we have added a statement to include the suggested scenario within the discussion.

Reviewer #2: In general, this was a well-written paper with largely well-designed experiments that should be easily accessible for a broad audience. I have only some relatively cosmetic suggestions for the authors.

Major comments:
1. Fig. 2B. Statistical analysis suggests that the p value is greater than 0.05 (therefore ‘not significant’), and the authors conclude that increase in toxin production was not due to increased colonization or carriage. That said, the difference in the spread of the data between the two groups is striking and it really looks like deleting spo0E results in reduced number of bacteria in the cecum., depending on the particular animal. Is it possible that increased toxin production *causes* a decreased C. difficile burden in a fraction of animals? In other words, what is the virulence/pathogenesis consequence of increased toxin production? Not requesting additional experiments here, just proposing a different interpretation of the results that the authors may or may not wish to address.	We agree that regardless of the p value, there are fewer CFU in the *spo0E* mutant than the parent strain-infected animals and this is likely because the early toxin production of the mutant kills the animals more rapidly than the bacteria can achieve high CFU. We have attempted to explain this in results section for Fig. 2.

2. Fig. 2, Table 1. If modeling predicts that Spo0E, RstA, and Spo0A form a complex, any idea why RstA did not copurify with Spo0A (was RstA present, but below the cutoff value in the LC-MS/MS data for the Spo0A co-IP)?	As mentioned for Rev1, Q4, we did find RstA sometimes in the Spo0A pulldowns, but it was below the cutoff for inclusion.

3. Consider moving the model figure in Fig. S7 to the main text as a new Fig. 5.	We have moved the model to the main text as suggested.

Reviewer #3: 1. Some insight into what the important interactions of Spo0E would strengthen the paper Ideally this would be point mutants that disrupt interaction with Spo0A or RtsA specifically but may be outside the scope. A less difficult experiment would be to construct a spo0E rstA double mutant to determine if it resembles a spo0E or rtsA phenotype. This could provide some insight to which interactions are more important.	We are now generating site-directed mutants of Spo0E in *C. difficile* to test interactions with Spo0A and RstA, as well as their functionality. However, these experiments are a major component of a new student’s thesis and are not progressing quickly enough for inclusion in the current study.	We have made the *spo0E rstA* double mutant and tested its sporulation and toxin expression, but the results led to more questions than answers. The *spo0E rstA* mutant has ∆*spo0E-*like toxin expression (very high), which is 50% greater than *rstA.* But, the double mutant has intermediate sporulation (~7%), compared to the low *rstA* (~2%) and ∆*spo0E* (~55%). Based on expression data and other mutants, there appear to be complex feedback effects from several other factors that modulate these phenotypes. Chasing down the mechanisms responsible for the phenotypes became a journey of its own that distracted from the aims of the current work, so we plan to publish that study separately.

2. The CO-IP mass spec experiments should be confirmed using another assay.

As outlined above, n addition to the Spo0E and Spo0A pulldowns and mass spec analyses, we now confirm by western blot analysis the interactions between Spo0E and RstA using antibodies to each of the proteins in the new Figure 4.

**Part III – Minor Issues: Editorial and Data Presentation Modifications**
Please use this section for editorial suggestions as well as relatively minor modifications of existing data that would enhance clarity.

Reviewer #1: Minor comments.
Could the authors comment on why the motility phenotype is so modest as compared to toxin production if both are thought to be mediated by RstA and SigD?	There are multiple other regulatory factors besides RstA and SigD that affect motility. Although RstA and SigD are negative and positive regulators of transcription of motility-associated genes, many of the proteins associated with motility are post-translationally controlled, which is not the case for the toxins.

Line 203. ‘Complement’ probably isn’t the best word choice, maybe state as the converse experiment?	We have amended the statement as suggested.

Reviewer #2: Minor comments:
1. Line 106. Please define “RRNPP” upon first use.	ok

2. Fig. 1B, 1D, Fig. 4. Consider eschewing the use of bar graphs and instead use scatter graphs (similar to Fig. 2B-D) so that the reader may appreciate the spread of data.	We have used both scatter and bar graphs to represent these data to provide a holistic view of the results.

3. The complementation data in Fig. S2 is very important. Consider moving Fig. S2A-B into Fig. 1.	We attempted to combine these figures but the resulting compilation exceeded the capacity of a page due to the separate control strains required for the complementation experiments, so we now include these data as a separate figure in the main text (now Fig 2).

Reviewer #3: 1. I would like to see the complementation data in the main text.	We now include these data as an additional figure in the body of the manuscript.

2. Do Spo0E homologs from other organisms complement either B. subtilis or C. difficile?
Other than our study, the only cross-complementation of Spo0E performed was in JBac 2007 189(7):2637-2645, wherein the authors over-expressed a *B. anthracis spo0E* in *B. subtilis* and demonstrated a decrease in sporulation frequency.

3. It would be helpful to know if the flag-tagged proteins are functional (do they complement mutants).	Yes, the tagged proteins are functional. We now clarify this in the text.
