# Supplementary material for: A conserved switch controls virulence, sporulation, and motility in C. difficile
Source: PLoS Pathog. 2024 May 13;20(5):e1012224. doi: 10.1371/journal.ppat.1012224 (PMC11115286; doi:10.1371/journal.ppat.1012224)
Supplement: Attachment — Submitted filename: Spo0E Response to Reviewers-2.docx [file ppat.1012224.s016.docx]

*Editor note:*

The newly added co-immunoprecipitation data greatly strengthened this interesting manuscript. The Reviewers and I are in agreement that the article should be accepted, but there is a request for very slight changes in a revised manuscript that can be easily addressed. It would be nice to include the silver-stained gel of the pull-downs into the supplemental (as Reviewer 3 notes, the methods described silver staining). Reviewer 3 also had questions about the band labeled as "RstA dimer" in the co-IP. For example, there is a band that may run at a similar size in the Spo0A western blot (but it is hard to tell with the MW markers shown).

In addition, to avoid confusion of readers who may be quickly glancing at the figures to indicate that the structures shown are AlphaFold models in the figure legends and figure legend title. Please change the title of Figure 5 so that it reads "C. difficile Spo0E is predicted to interact with functional domains of Spo0A and RstA." Similarly, for supplemental figure 3, please revised the legend title to read "Predicted C. difficile Spo0E interactions with Spo0A and RstA."

 As requested:

1) We have added the silver-stained gel of the pulldowns (in Figure 4).	The RstA dimer/size are now labelled on the gel.

2) The figure legends for AlphaFold model figures (Fig 5 and S3) have been amended as suggested.

Reviewer Comments (if any, and for reference):

Reviewer's Responses to Questions

**Part I - Summary**
Please use this section to discuss strengths/weaknesses of study, novelty/significance, general execution and scholarship.

Reviewer #1: (No Response)

Reviewer #3: In the initial review I raised two concerns. The first was a confirmation of the interaction between SpoOE and RstA which was provided and convincing. They were unable to confirm with genetics that this interaction is biologically relevant. Otherwise they have addresssed the majority of the concerns well.

**Part II – Major Issues: Key Experiments Required for Acceptance**
Please use this section to detail the key new experiments or modifications of existing experiments that should be absolutely required to validate study conclusions.

Generally, there should be no more than 3 such required experiments or major modifications for a "Major Revision" recommendation. If more than 3 experiments are necessary to validate the study conclusions, then you are encouraged to recommend "Reject".

Reviewer #1: (No Response)

Reviewer #3: (No Response)

**Part III – Minor Issues: Editorial and Data Presentation Modifications**
Please use this section for editorial suggestions as well as relatively minor modifications of existing data that would enhance clarity.

Reviewer #1: (No Response)

Reviewer #3: I am not convinced based on what the authors have written that what is labeled as RtsA dimer is really a dimer. Is it possible that this has not entered the gel (there are not standards labeled above the "dimer)? If looked at closely there are "dimer" bands present in the Eluate of all 3 samples. It would be beneficial to include the silver stain in the supplemental figures as it is mentioned in the methods but I did not see this in the main or supplemental figures.
